# Long non‐coding RNA HOXC‐AS1 exerts its oncogenic effects in esophageal squamous cell carcinoma by interaction with IGF2BP2 to stabilize SIRT1 expression

**DOI:** 10.1002/jcla.24801

**Published:** 2022-12-12

**Authors:** Zhengwu Yang, Junhu Wan, Liwei Ma, Zhuofang Li, Ruotong Yang, Haijun Yang, Junkuo Li, Fuyou Zhou, Liang Ming

**Affiliations:** ^1^ Department of Clinical Laboratory The First Affiliated Hospital of Zhengzhou University, and the Key Clinical Laboratory of Henan Province Henan China; ^2^ Henan Key Medical Laboratory of Precise Prevention and Treatment of Esophageal Cancer Anyang China; ^3^ Department of Pathology Anyang Cancer Hospital, The Forth Affiliated Hospital of Henan University of Science and Technology Henan China; ^4^ Thoracic Department Anyang Tumor Hospital, Henan Key Medical Laboratory of Precise Prevention and Treatment of Esophageal Cancer Anyang China

**Keywords:** ESCC, HOXC‐AS1, IGF2BP2, RNA binding protein, SIRT1

## Abstract

**Background:**

Long non‐coding RNA HOXC cluster antisense RNA 1 (HOXC‐AS1) is a novel lncRNA whose cancer‐promoting effect in gastric cancer and nasopharyngeal carcinoma has already been demonstrated. However, its functions in esophageal squamous cell carcinoma (ESCC) remains unknown. LncRNAs can interact with RNA‐binding proteins (RBPs) and affect gene expression levels through post‐transcriptional regulation. Insulin‐like growth factor 2 mRNA‐binding protein 2 (IGF2BP2) is a widely studied RBP, and sirtuin 1 also known as SIRT1 has been reported to be involved in cancer progression.

**Methods:**

Establishment of in vivo models, HE and immunohistochemistry staining verified the oncogenic effect of HOXC‐AS1. The interaction relationship between HOXC‐AS1, IGF2BP2 and SIRT1 was verified by RNA pulldown and RNA immunoprecipitation (RIP) assay. Relative expression and stability changes of genes were detected by qPCR and actinomycin D experiments. Finally, the effect of HOXC‐AS1‐IGF2BP2‐SIRT1 axis on ESCC was verified by rescue experiments.

**Results:**

HOXC‐AS1 is highly expressed in ESCC cells and plays oncogenic effects in vivo. qPCR showed the positive relationship between HOXC‐AS1 and SIRT1 following HOXC‐AS1 knockdown or overexpression. RNA‐pulldown, mass spectrometry and RIP assay demonstrated that IGF2BP2 is an RBP downstream of HOXC‐AS1. Then, RIP and qPCR showed that IGF2BP2 could bind to SIRT1 mRNA and knockdown IGF2BP2 resulted in decreased SIRT1 mRNA level. Finally, a series of rescue assay showed that the HOXC‐AS1‐IGF2BP2‐SIRT1 axis can affect the function of ESCC.

**Conclusion:**

LncRNA HOXC‐AS1 acts as an oncogenic role in ESCC, which impacts ESCC progression by interaction with IGF2BP2 to stabilize SIRT1 expression.

## Graphical abstract

Long non‐coding RNA HOXC‐AS1 exerts its oncogenic effects in esophageal squamous cell carcinoma by interaction with IGF2BP2 to stabilize SIRT1 mRNA expression.

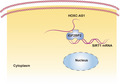

## 1 INTRODUCTION

Esophageal carcinoma (EC) is the seventh most common malignancy tumor all over the world, causing over 400 thousand deaths worldwide annually. Esophageal squamous cell carcinoma (ESCC) is the most common histological subtype.^1^, ^2^ Although tremendous advances being made in the surgical and preoperative radiotherapy and chemotherapy for ESCC in recent years, but most ESCC patients are diagnosed at an advanced stage and cannot undergo surgical resection. High mortality and low survival rates of this disease are great challenges for human health. Thus, there is a compelling need to find predictive biomarkers and therapeutic targets for the early diagnosis and treatment of ESCC.

Increasing clues suggest that many long non‐coding RNAs (lncRNAs) can participate in tumor advancement by regulating coding gene expression both transcriptionally and post‐transcriptionally. For instance, Wang et al. found that upregulated LINC00482 in bladder cancer can increase recruitment of the MMP15 promoter region by binding the transcription factor FOXA1.^3^ Ma et al. demonstrated that lncRNA NEAT1 can inhibit GADD45A expression through recruiting BRG1, and then H3K27me3 and H3K4me3 were installed at the promoter region of GADD45A thereby promoting gastric cancer progression.^4^ LncRNAs can also interact with RNA‐binding proteins (RBPs) by acting as molecular scaffolds and affect gene expression levels through post‐transcriptional regulation. Wang et al. illustrated that lncRNA FIRRE can physically interact with RNA binding protein PTBP1 to stabilize BECN1 thereby promoting autophagy in colorectal cancer.^5^ Huang et al. found that linc01305 can facilitate ESCC progression through interacting with IGF2BP2 and IGF2BP3 to maintain HTR3A mRNA expression.^6^ LncRNA HOXC‐AS1 plays a cancer‐promoting role in a variety of tumors,^7^, ^8^, ^9^ but its functions in ESCC still remain unknow.

Here, our research explores the mechanism by which HOXC‐AS1 promotes ESCC progression through post‐transcriptional regulation, provides a new target for the diagnosis and therapy of ESCC patients.

## 2 MATERIALS AND METHODS

### 2.1 Cell culture and transfection

The human esophageal squamous carcinoma cell lines KYSE30, Eca109 and human normal esophageal epithelial cells HEEC were bought from the American Type Culture Collection (ATCC), and cultured in 37°C with 5% CO_2_. RPMI 1640 medium (Yuanpei Biotechnology Co., Ltd) was used for cell culture with 10% fetal bovine serum (Cell‐Box Biological products Trading Co., Ltd) and 1% antibiotics. HOXC‐AS1 was knockdown or overexpression by using the HOXC‐AS1 shRNA or full‐length sequences‐packaged lentivirus. SIRT1 plasmid was used to overexpress SIRT1 level. The lentiviral vector systems and overexpression plasmids were all purchased from Genechem Co.,Ltd. Puromycin was used to select the transfected cells for 1 week. The knockdown of IGF2BP2 was implemented by transfecting siRNA (Sangon Bioengineering Co., Ltd). All operations were performed according to the manufacturer's instructions. The shRNA and siRNA sequences are showed in the Table S1.

### 2.2 Xenograft models

KYSE30 cells (2 × 10^6^) expressing Lv‐shNC or Lv‐shHOXC‐AS1 were subcutaneously injected into the back of 3‐ to 4‐week‐old female BALB/c nu/nu mice (4 mice per group). Tumor volume was measured by vernier caliper every 4 days. 24 days after injection, the mice were euthanized, the tumor weight and volume were measured. Finally, the tumors were embedded in paraffin and sectioned for HE or IHC staining.

### 2.3 RNA isolation and quantitative RT‐PCR

Total RNA was extracted with TRIzol reagent (Takara) and reverse transcribed into cDNA by PrimeScript™ RT reagent Kit (Takara). qPCR was accomplished using TB green Premix Ex Taq™ II kit (Takara) on LightCycler480 system (Roche, Swiss). Relative expression level of target gene was normalized by actin and calculated with the 2^−∆∆Ct^ method. The primers sequences used were as follows: HOXC‐AS1: forward primer 5′ ‐(GCCAGCTTGAAGAAGTGTAGGAGAG)‐3′, reverse primer 5′ ‐(GGAAGTGTCGCAGAGATGGAGTTG)‐3′. IGF2BP2: forward primer 5′ ‐(ATCGGGAGCAAACCAAAGACCATC)‐3′, reverse primer 5′ ‐(CTGGCAAACCTGGCTGACCTTC)‐3′. SIRT1: forward primer 5′ ‐(TTGCTCTTGTTGTCCAGGCTGAAG)‐3′, reverse primer 5′ ‐(GAGGCAGGAGAATCGCTTGAACC)‐3′. Actin, forward primer 5′ ‐(CCTGGCACCCAGCACAAT)‐3′, reverse primer 5′ ‐(GGGCCGGACTCGTCATAC)‐3′.

### 2.4 RNA immunoprecipitation (RIP)

To verify the interaction of IGF2BP2 protein with HOXC‐AS1 and SIRT1 RNA, an RIP assay was accomplished according to the manufacturer's protocol of the RNA Immunoprecipitation Kit (Cat#P0102, Geneseed Biotech). RNAs and proteins were isolated and then detected by qPCR and western blot.

### 2.5 RNA pull‐down

Full‐length biotin‐labeled HOXC‐AS1 and antisense sequences probe were synthesized by Gemma Gene. Then RNA pull‐down assays were accomplished according to the manufacturer's instructions of the Magnetic RNA‐Protein Pull‐Down Kit (Cat#20164, Thermo Scientific). The captured proteins of sense and antisense probe were detected by mass spectrometry (APTBIO) and western blot.

### 2.6 Protein extraction and western blotting

Cells were lysed on ice using RIPA (New cell & Molecular Biotech Co., Ltd). The protein concentration was detected using the BCA protein quantification kit (Beyotime, Shanghia, China). Protein samples were separated on 10% SDS‐PAGE gels (Epizyme Biomedical Technology Co., Ltd) and then transferred onto PVDF membrane. After incubated with 5% nonfat milk for 1 h, the membrane was incubated with primary antibodies overnight at 4°C. After washed for three times, the HRP‐conjugated secondary antibodies were added to the membranes for incubation for 1 h at room temperature. The blots were visualized with ECL kit (Epizyme Biomedical Technology Co., Ltd). The primary antibodies IGF2BP2, SIRT1 and GAPDH were bought from Proteintech (Wuhan, China).

### 2.7 Actinomycin D and RNA stability assay

Transfected cells were treated with actinomycin D at a concentration of 5 μg/ml. Total RNA was extracted from cells every 2 h from 0 h to 10 h. HOXC‐AS1 overexpression and IGF2BP2 knockdown or in together were handled with actinomycin D (7.5 μg/ml) for 60 min and then extracted total RNA. The total RNA was reverse transcribed into cDNA and qPCR was applied for quantitative detection. Actin served as an internal control.

### 2.8 EdU assay

A 100 μl cell suspension (2 × 10^3^ cells) was seeded into 96‐well plates and incubated overnight. 50 μM EdU reagent (Beyotime technology, Shanghai) was added and incubated for 2 h. Then cells were fixed with formaldehyde and the nuclear was stained with Hoechst. Finally, observed and photographed with fluorescence microscope.

### 2.9 Transwell migration assay

Cell migration capacity was detected by using the 24‐transwell migration chambers (Costar). 3 × 10^4^ cells were seeded in upper chamber with 200 μl serum‐free medium, and 600 μl 15% serum medium was added into the lower chamber. 48 h later, cells were fixed with formaldehyde and then dyed with crystal violet. The migrated cells were photographed under a microscope and recorded.

### 2.10 Colony formation

2 × 10^3^ cells were seeded in six‐well plate and incubated in RPMI 1640 medium with 10% fetal bovine serum for 10 days, then fixed in paraformaldehyde and stained with crystal violet for 30 min. the colonies were photographed and counted.

### 2.11 Statistical analysis

The experimental data was collected and statistically analyzed by using GraphPad Prism 8. Comparisons in two groups were made using the unpaired Student's t‐test. Correlations between HOXC‐AS1 and SIRT1 gene expression were analyzed by spearman correlation analysis. All data were recorded as Mean ± SD, and *p* < 0.05 was considered significant.

## 3 RESULTS

### 3.1 HOXC‐AS1 is highly expressed in ESCC cells and plays oncogenic effects in vivo

**FIGURE 1:**
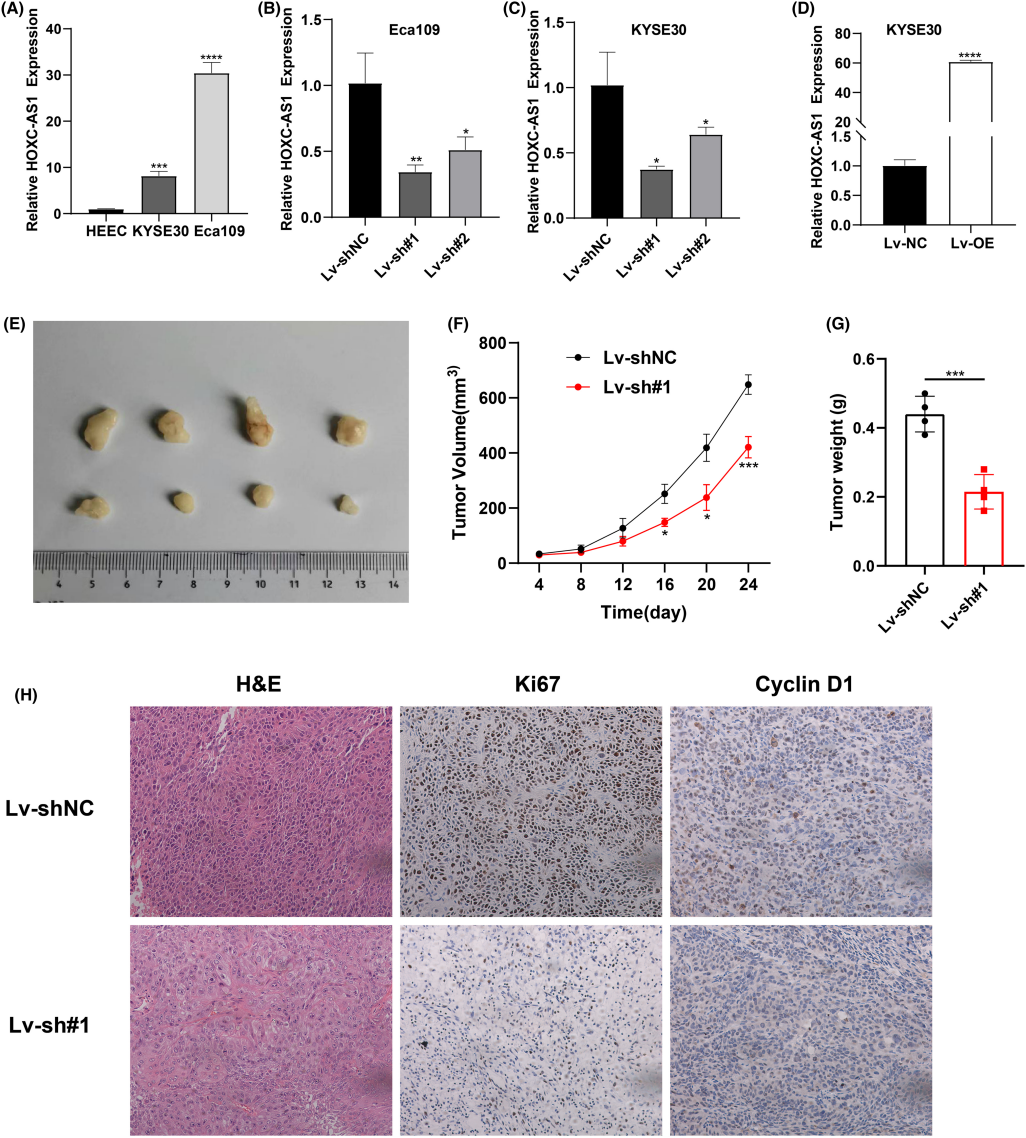
HOXC‐AS1 is highly expressed in ESCC cells and plays oncogenic effects in vivo. (A) Relative expression of HOXC‐AS1 in ESCC cell lines. (B) knockdown efficiency of HOXC‐AS1 in Eca109 cell line. (C–D) knockdown and overexpression efficiency of HOXC‐AS1 in KYSE30 cell line. (E) HOXC‐AS1 knockdown in KYSE30 inhibited tumor growth in vivo. (F–G) Statistics analysis of tumor volume and tumor weight in subcutaneous implant mouse models. (H) HE and IHC images of tumor tissue in both groups. *p < 0.05, **p < 0.01, ***p < 0.001, ****p < 0.0001

Long non‐coding RNA HOXC‐AS1 has already been elucidated to be overexpressed in gastric cancer and nasopharyngeal carcinoma.^8^, ^9^ However, its role in ESCC is still unclear. Firstly, we used qPCR to detect the relative expression of HOXC‐AS1. As shown in Figure 1A, HOXC‐AS1 was significantly overexpressed in esophageal squamous cell carcinoma cell lines KYSE30 and Eca109 than that in human normal esophageal epithelial cells (HEEC). Then*, we* constructed stable HOXC‐AS1 knockdown and overexpression cell lines and the transfection efficiency was confirmed by qPCR (Figure 1B–D). After that, the effect of HOXC‐AS1 in tumorigenesis in vivo was explored. As shown in Figure 1 E–G, the tumor size, weight and volume were significantly lower after HOXC‐AS1 knockdown. Then, the tumor tissue was stained by Hematoxylin–Eosin (H&E) and immunohistochemistry (IHC). As shown in Figure 1H, HOXC‐AS1 knockdown decreased the positive rate of the cell proliferation marker Ki67 and the cell cycle marker Cyclin D1. All the result suggested that HOXC‐AS1 plays an oncogenic effect in ESCC.

### 3.2 HOXC‐AS1 can stabilize SIRT1 mRNA expression

**FIGURE 2:**
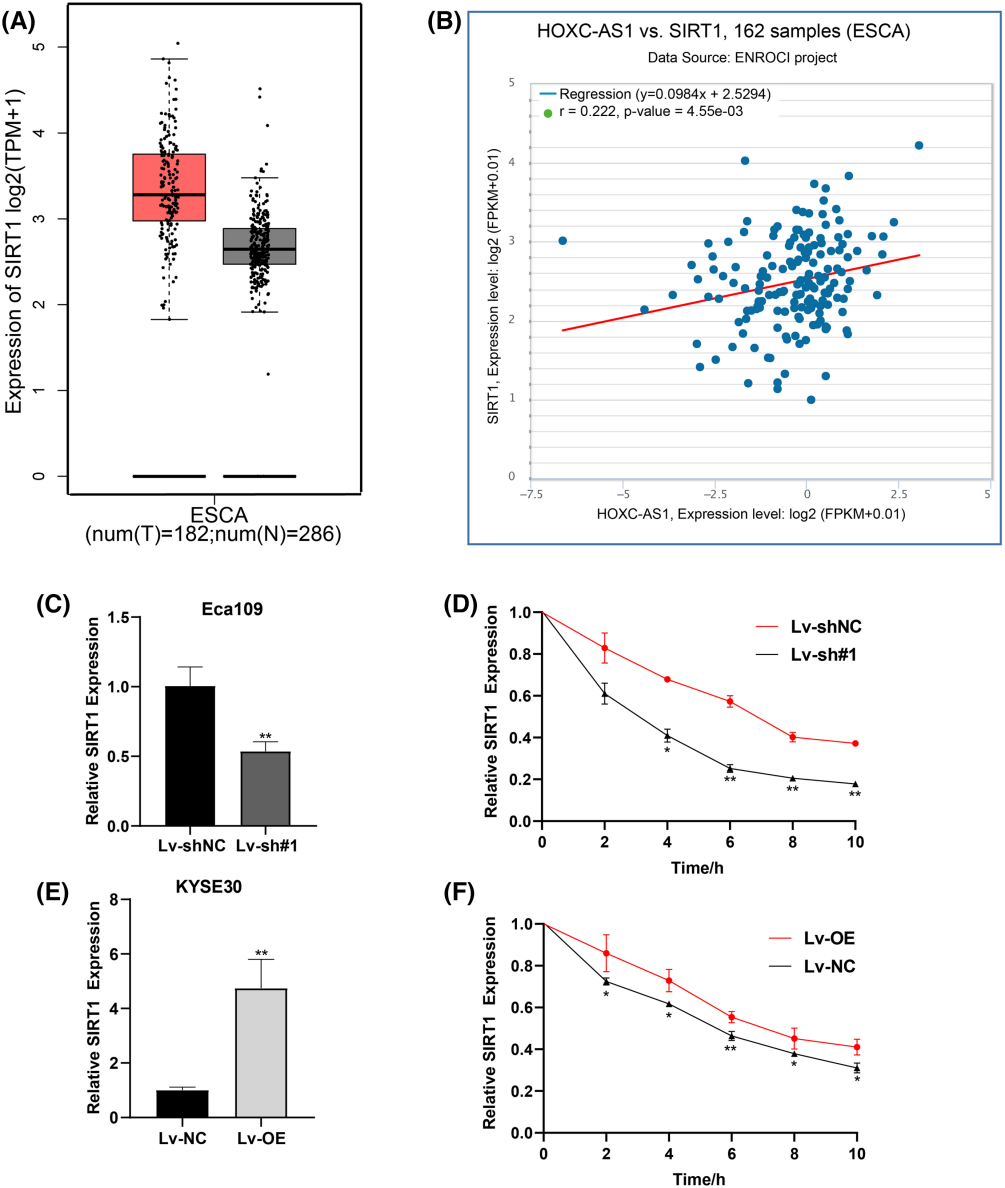
HOXC‐AS1 can affect the SIRT1 mRNA. (A) Relative SIRT1 expression of tumor and normal in GEPIA database. (B) HOXC‐AS1 and SIRT1 expression are positively correlated in starbase database. (C–F) SIRT1 mRNA expression and stability were detected when HOXC‐AS1 was knockdown in Eca109 and KYSE150. *p < 0.05, **p < 0.01

Sirtuin‐1 (SIRT1) is an important NAD + ‐dependent histone deacetylase in mammals, and participates many biological processes in cancer.^10^, ^11^ Here, we explored the relationship between HOXC‐AS1 and SIRT1. By using the GEPIA database (http://gepia2.cancer‐pku.cn/). We found that SIRT1 expressed significant highly in ESCA samples than that in normal samples (Figure 2A). The RNA–RNA Coexpression module of starbase database (starbase.sysu.edu.cn) showed a positive relationship between SIRT1 and HOXC‐AS1 expression in ESCA samples (Figure 2B). Then we detected the expression of SIRT1 in HOXC‐AS1 knockdown as well as overexpress cell lines, and observed the effect of HOXC‐AS1 on SIRT1 mRNA using Actinomycin D assay. As shown in Figure 2C–F, the relative expression and the stability of SIRT1 was diminished in HOXC‐AS1 knockdown cell line. While, the situation was reversed when HOXC‐AS1 was overexpressed. The above results indicate that HOXC‐AS1 can stabilize SIRT1 mRNA expression.

### 3.3 HOXC‐AS1 can interact with RNA‐binding protein IGF2BP2

**FIGURE 3:**
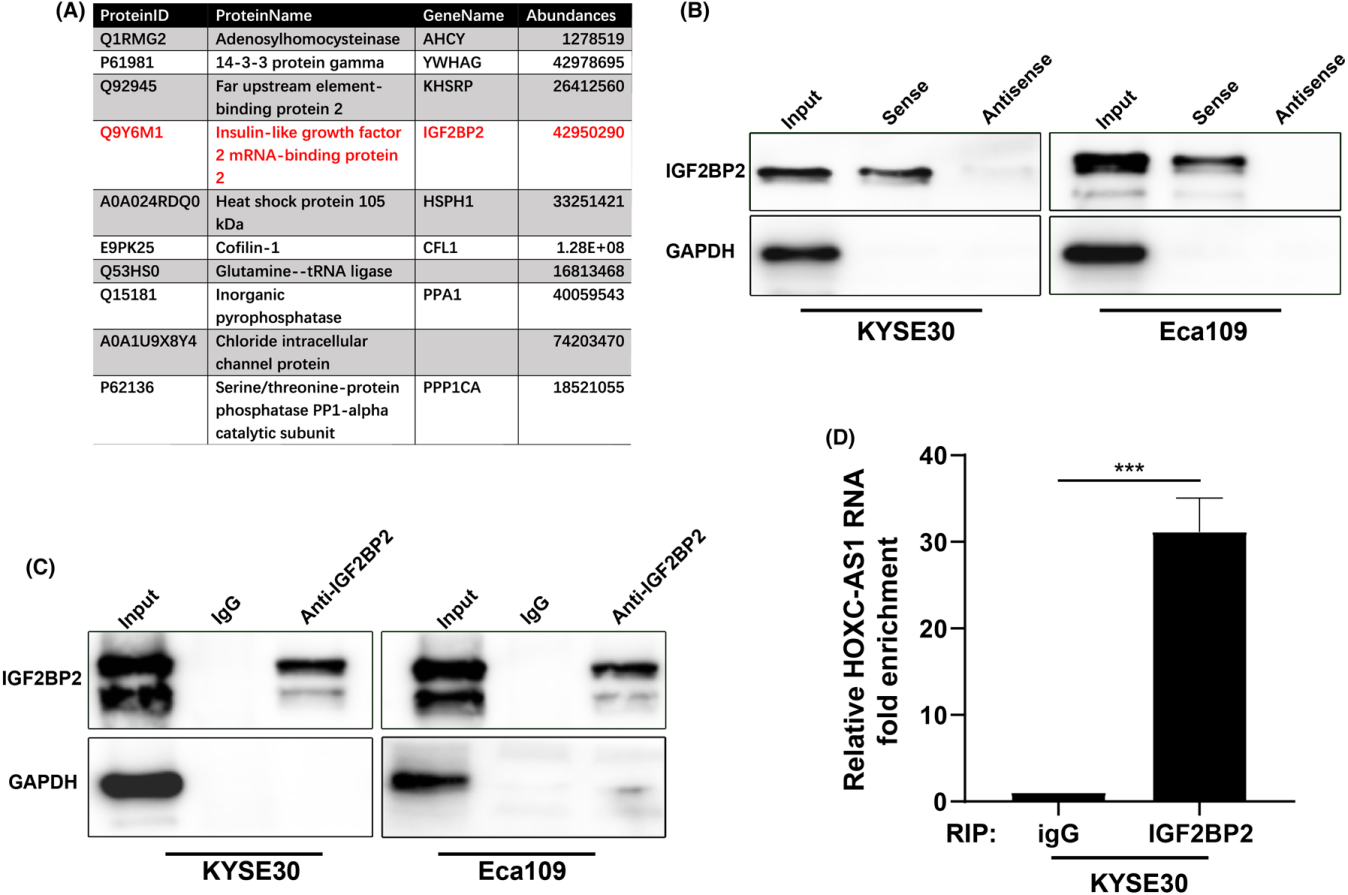
HOXC‐AS1 directly interacts with RNA‐binding protein IGF2BP2. (A) Mass spectrum results showed IGF2BP2 can bind to HOXC‐AS1 sense group. (B), Western blot showed that the sense of HOXC‐AS1 but not antisense captured IGF2BP2. (C) The efficiency of immunoprecipitation was texted by western blotting. (D) qPCR showed the anti‐IGF2BP2 enriched more HOXC‐AS1 RNA than anti‐igG. *p < 0.05, **p < 0.01, ***p < 0.001

To further investigate the mechanism by which HOXC‐AS1 regulates SIRT1, we designed full‐length biotin‐labeled HOXC‐AS1 sense and antisense sequences probes, then, the RNA pull‐down assay was been performed. Mass spectrometry results of the sense group showed that IGF2BP2, which has already been reported to be involved in the regulation of mRNA stability,^6^, ^12^ could bind to HOXC‐AS1 (Figure 3A). Then western blot was conducted to further demonstrate the interaction of HOXC‐AS1 and IGF2BP2, the results showed that HOXC‐AS1 but not antisense captured IGF2BP2 (Figure 3B). Next, the binding of IGF2BP2 with HOXC‐AS1 was confirmed by RIP assay. The efficiency of immunoprecipitation was texted by western blotting (Figure 3C). Then, the immunoprecipitated RNAs was analyzed by qPCR. As shown in Figure 3D, the anti‐IGF2BP2 enriched more HOXC‐AS1 RNA than anti‐IgG. These results confirmed the interaction between IGF2BP2 protein and HOXC‐AS1 RNA.

### 3.4 HOXC‐AS1 enhances SIRT1 mRNA stability in a IGF2BP2‐madiated manner

**FIGURE 4:**
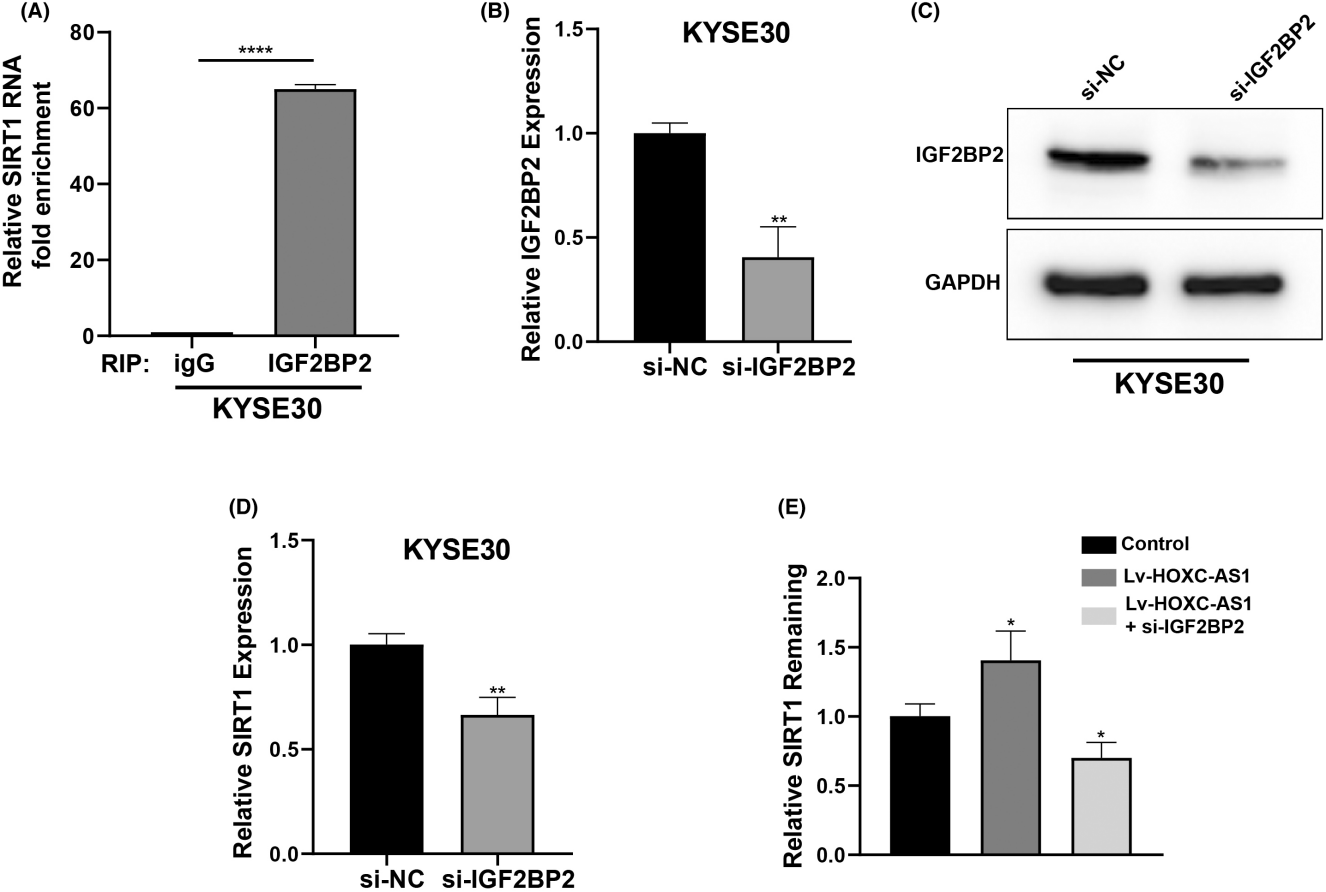
HOXC‐AS1 enhances SIRT1 mRNA stability in a IGF2BP2‐madiated manner. (A) RIP assay showed the anti‐IGF2BP2 group enriched more SIRT1 mRNA than anti‐igG group. (B–C), The efficiency of IGF2BP2 knockdown in KYSE30 cell line measured by qPCR and western blot. (D) The relative SIRT1 expression was detected by qPCR in si‐NC and si‐IGF2BP2 group. (E) Relative SIRT1 remaining after actinomycin D treatment for 60 min.*p < 0.05, **p < 0.01 ***p < 0.001, ****p < 0.0001

IGF2BP2 has already been reported as an RNA‐binding protein related to mRNA stability, we then discovered whether the association between HOXC‐AS1 and IGF2BP2 may have impact on SIRT1 mRNA. SIRT1 was predicted as a target mRNA of IGF2BP2 protein using starbase database. The RIP assay was performed to verify the interaction between IGF2BP2 and SIRT1. As shown in Figure 4A, the SIRT1 mRNA was strongly enriched in anti‐IGF2BP2 group than anti‐IgG group. We designed siRNA to knockdown IGF2BP2, the mRNA and protein level of IGF2BP2 were diminished significantly (Figure 4B,C). The relative SIRT1 mRNA expression was restrained after IGF2BP2 knockdown. (Figure 4D). These results suggested that SIRT1 mRNA is a target mRNA of IGF2BP2 protein. Finally, we transfected KYSE30 cells with Lv‐HOXC‐AS1 and si‐IGF2BP2 or in together, and cells were treated with actinomycin D for 60 min to observe the half‐life of SIRT1 mRNA. As shown in Figure 4E, compared to control group, HOXC‐AS1 overexpression enhanced the stability of SIRT1 mRNA, while IGF2BP2 knockdown could rescued the stabilizing effect of HOXC‐AS1 on SIRT1 mRNA. These results indicated that HOXC‐AS1 stabilized SIRT1 mRNA in an IGF2BP2‐madiated manner.

### 3.5 HOXC‐AS1 exerts its oncogenic effects in esophageal squamous cells through elevating SIRT1 expression

**FIGURE 5:**
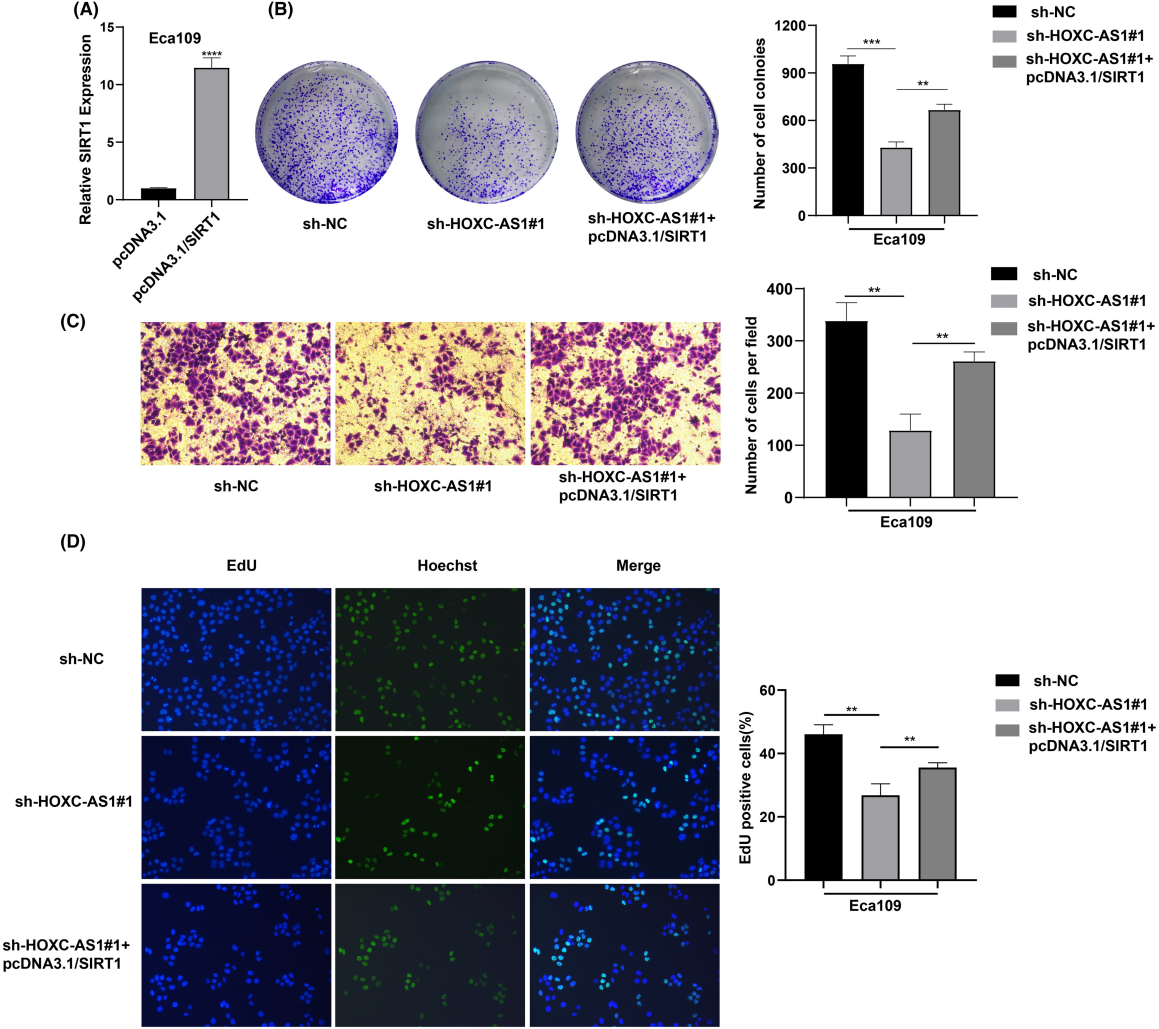
HOXC‐AS1 exerts its oncogenic effects in ESCC through elevating SIRT1 expression. (A) Overexpression efficiency of SIRT1 in Eca109 cell line detected by qPCR. (B) Colony formation assay for the transfected Eca109 cells. (C) Transwell assay was performed to detect the migration of transfected Eca109 cells. (D) EdU assay was performed to detect cell proliferation ability of transfected Eca109 cells. *p < 0.05, **p < 0.01, ***p < 0.001, ****p < 0.0001

Since HOXC‐AS1 functioned in SIRT1 mRNA stability, we investigated whether HOXC‐AS1 was involved in the development of esophageal carcinoma via SIRT1. Firstly, we transfected SIRT1 overexpress plasmid in Eca109 and detected the overexpression efficiency by using qPCR (Figure 5A). Then we performed a series of functional assays. Colony formation assay demonstrated that SIRT1 overexpression rescued the attenuation of cell colony‐forming ability caused by HOXC‐AS1 knockdown (Figure 5B). Transwell and EdU assay confirmed that the up‐regulation of SIRT1 counteracted the attenuation of cell migration and proliferation caused by HOXC‐AS1 knockdown (Figure 5C,D). In a word, HOXC‐AS1 can promote esophageal squamous cells function in a SIRT1 dependent manner.

## 4 DISCUSSION

In recent years, overwhelming evidence has demonstrated that lncRNAs has an important role in human diseases, especially in cancers.^13^ HOXC‐AS1 is a novel lncRNA which has already been discovered to play an oncogenic role in gastric cancer and nasopharyngeal carcinoma. It can promote tumor progression by sponging miRNA to function as a competing endogenous RNA (ceRNA), and also can interact with c‐MYC to form a HOXC‐AS1‐MYC feed‐forward loop to exacerbate tumor progress in GC.^7^, ^8^, ^9^ In this study, we firstly report the oncogenic effects of HOXC‐AS1 in ESCC. HOXC‐AS1 is highly expressed in ESCC cell lines and promotes tumor growth in vivo. This indicates that HOXC‐AS1 is positively associated with the progression of ESCC.

RNA‐binding proteins can act as an important regulator of lncRNAs in many kinds of cancers.^14^ The human insulin‐like growth factor 2 mRNA binding proteins 2 (IGF2BP2) was found that it can bind RNAs and impact on their transcript target through post‐transcriptional regulation ways in tumor development.^15^, ^16^ For example, Huang et al. found that linc01305 can facilitate ESCC progression through interacting with IGF2BP2 and IGF2BP3 to maintain HTR3A mRNA expression.^6^ Shen et al. demonstrated that LINC01559 can promote gastric cancer process by recruiting IGF2BP2 to elevate ZEB1 mRNA.^17^ Besides, IGF2BP2 is an N6‐methyladenosine (m6A) reader which can stabilize mRNA in an m6A‐dependent manner. For instance, Hou et al. found that LINC00460 can promote colorectal cancer proliferation and metastasis by interaction with IGF2BP2 and DHX9 to regulate HMGA1 expression in a m6A modification dependent manner.^18^ Pu et al. demonstrated that IGF2BP2 can recognize and bind to the m6A site on FEN1 mRNA and enhanced FEN1 mRNA stability to exert its carcinogenic effect in liver cancer.^19^ Sirtuin‐1 (SIRT1) which known as a histone deacetylase has already been demonstrated to be involved in autophagy, apoptosis, senescence, genome stability maintenance in cancer through multiple approaches. Wang et al. found that lncRNA H19 can sponge miR‐194‐5p to modulate SIRT1 expression thereby inducing autophagy.^20^ Yousafzai et al. described that SIRT1 can stabilize XRRC1 by inhibiting its ubiquitination‐dependent degradation and thus promote chemoresistance in lung cancer.^21^ Because both IGF2BP2 and SIRT1 play an important role in tumor, we hypothesize that their mutual regulation also can affect the occurrence and development of ESCC, lncRNA HOXC‐AS1 acts as a molecular scaffold in this progress.

In our study, we found that HOXC‐AS1 is positively correlated with the expression of SIRT1 mRNA, RNA pulldown and RIP assay showed that HOXC‐AS1 can bind to the RNA‐binding protein IGF2BP2. IGF2BP2 has already been reported to regulate mRNA stability, our subsequent experiments demonstrated that it can bind to and enhance SIRT1 mRNA stability. A HOXC‐AS1‐IGF2BP2‐SIRT1 axis in ESCC regulation was emerging. Finally, we performed rescue assay and demonstrated that HOXC‐AS1 exerts its oncogenic effects in ESCC through elevating SIRT1 expression.

However, our work still has some limitations. For example, IGF2BP2 as a widely studied RBP and we found that it can stabilize SIRT1 mRNA in ESCC, we conjecture that HOXC‐AS1 acts as a molecular scaffold in it to connect IGF2BP2 and SIRT1. But the mechanism still requires further investigation. Besides, the mechanism by which SIRT1 regulates ESCC has not been elucidated.

In summary, our research provides evidence that lncRNA HOXC‐AS1 promotes ESCC development by interacting with RNA‐binding protein IGF2BP2 to stabilize SIRT1 mRNA expression, indicates that HOXC‐AS1‐IGF2BP2‐SIRT1 axis may serve as a potential prognostic marker and therapeutic target for ESCC.

## AUTHOR CONTRIBUTIONS

Zhengwu Yang and Junhu Wan designed and performed in vitro experiments, Liwei Ma, Zhuofang Li and Ruotong Yang performed in vivo animal experiments, Haijun Yang, Junkuo Li helped with statistical analysis and picture production, Zhengwu Yang and Junhu Wan conceived and wrote the manuscript, Liang Ming and Fuyou Zhou approved the final manuscript.

## FUNDING INFORMATION

This study was supported by grants from the National Natural Science Foundation of China (82172351, 82173018), Key scientific research project plan of colleges and universities in Henan Province (22A320074) and Natural Science Foundation of Henan Province (212300410395), Medical Science and Technology Research Project of Henan Province (SBGJ202102139 and SBGJ202102131), the Medical Science and Technology Provincial and Ministerial Co‐construction Project of Henan province (Grant No. SBGJ202102131, SBGJ202102133), the Young and Middle‐aged Health Science and Technology Innovation Talents Project of Henan province (Grant No. YXKC2021036).

## CONFLICT OF INTEREST

The authors have no relevant financial or non‐financial interests to disclose.

## Supporting information


Table S1

## Data Availability

All data used to support the findings of this study are available from the corresponding author upon request.
