# Supplementary material for: Long non‐coding RNA HOXC‐AS1 exerts its oncogenic effects in esophageal squamous cell carcinoma by interaction with IGF2BP2 to stabilize SIRT1 expression
Source: J Clin Lab Anal. 2022 Dec 12;37(1):e24801. doi: 10.1002/jcla.24801 (PMC9833966; doi:10.1002/jcla.24801)
Supplement: Supplementary file 1 — Table S1 [file JCLA-37-e24801-s001.docx]

Table S1. Sequences of shRNA and siRNA

| **NO.** | **5’** | **STEM** | **Loop** | **STEM** | **3’** |
| --- | --- | --- | --- | --- | --- |
| Lv-sh#1 | Ccgg | GGAGAGATCTACACAACTTAA | CTCGAG | TTAAGTTGTGTAGATCTCTCC | TTTTTg |
| Lv-sh#2 | Ccgg | GGCTCCTAGCTCATCTGAGAA | CTCGAG | TTCTCAGATGAGCTAGGAGCC | TTTTTg |
| Lv-shNC | Ccgg | TTCTCCGAACGTGTCACGT | CTCGAG | ACGTGACGTTCGGAGAA | TTTTTg |
| si-IGF2BP2 | Sense CGGAUCUUUGGGAAACUGAAATT  Antisense UUUCAGUUUCCCAAAGAUCCGTT | | | | |
| si-NC | Sense UUC UCC GAA CGU GUC ACG UTT  Antisense ACG UGA CAC GUU CGG AGA ATT | | | | |
